# Seasonal variation in objectively measured physical activity, sedentary time, cardio-respiratory fitness and sleep duration among 8–11 year-old Danish children: a repeated-measures study

**DOI:** 10.1186/1471-2458-13-808

**Published:** 2013-09-08

**Authors:** Mads F Hjorth, Jean-Philippe Chaput, Kim Michaelsen, Arne Astrup, Inge Tetens, Anders Sjödin

**Affiliations:** 1 Department of Nutrition, Exercise and Sports, Faculty of Science, University of Copenhagen, Rolighedsvej 30, DK-1958 Frederiksberg C, Copenhagen, Denmark; 2 Healthy Active Living and Obesity Research Group, Children’s Hospital of Eastern Ontario Research Institute, Ottawa, Ontario, Canada; 3 Division of Nutrition, DTU Food, National Food Institute, Technical University of Denmark, Copenhagen, Denmark

**Keywords:** Physical activity, Sedentary behaviour, Cardio-respiratory fitness, Sleep duration, Children, Season

## Abstract

**Background:**

Understanding fluctuations in lifestyle indicators is important to identify relevant time periods to intervene in order to promote a healthy lifestyle; however, objective assessment of multiple lifestyle indicators has never been done using a repeated-measures design. The primary aim was, therefore, to examine between-season and within-week variation in physical activity, sedentary behaviour, cardio-respiratory fitness and sleep duration among 8–11 year-old children.

**Methods:**

A total of 1021 children from nine Danish schools were invited to participate and 834 accepted. Due to missing data, 730 children were included in the current analytical sample. An accelerometer was worn for 7 days and 8 nights during autumn, winter and spring, from which physical activity, sedentary time and sleep duration were measured. Cardio-respiratory fitness was assessed using a 10-min intermittent running test.

**Results:**

The children had 5% more sedentary time, 23% less time in moderate-to-vigorous physical activity and 2% longer sleep duration during winter compared to spring and cardio-respiratory fitness was 4% higher during spring compared to autumn (P < 0.001). Sedentary time was higher and total physical activity, moderate-to-vigorous physical activity and sleep duration (boys only) were lower during weekends at all seasons (P ≤ 0.01). Intraclass correlation coefficients between seasons ranged from 0.47-0.74, leaving 45-78% to seasonal variation.

**Conclusions:**

Overall, sedentary time was higher and physical activity lower during winter and during weekends. The most accurate and unbiased estimates of physical activity came from autumn; however, the considerable intra-individual variation suggests that a single measurement may not adequately characterise children’s habitual sleep and activity.

## Background

The high prevalence of overweight and obesity among children in most parts of the world is a well-documented fact. Lower cardio-respiratory fitness (CRF) [1], short sleep duration [2], low levels of physical activity and high levels of sedentary behaviour [3] have been linked to the development of obesity and cardio-metabolic risk. As the reliability and validity of estimates of habitual physical activity [4] and sleep duration [5] is questionable when self-reported using questionnaires, accelerometers have become the preferred method of choice to measure physical activity and sedentary time; in recent years they have become an important method in sleep research [6]. Although the use of objective methods has increased in recent years, the majority of studies rely on single point measurements to characterise habitual physical activity, sedentary time, sleep duration and CRF. To the best of our knowledge, none have examined seasonal variation in accelerometer determined sleep duration or sedentary behaviour of children using a repeated-measures design, and only few studies have examined seasonal variation in physical activity of children using a repeated-measures design [7-10].

Collectively, objective assessment of seasonal pattern in multiple lifestyle indicators such as sleep, physical activity and sedentary behaviour across the year has never been done using a repeated-measures design. Given that the weather changes considerably across the year in northern Europe, it could help to put existing literature into context and identify relevant time periods to intervene in order to promote a healthy lifestyle. Furthermore, a single measure of physical activity, from a random time during the year, to quantify a relationship with obesity or cardio-metabolic risk, can lead to random error if large differences exist between seasons. However, when repeated measurements of the exposure variable (e.g. physical activity) are collected, the intra-class correlation coefficient (ICC), a measure of reliability, can be calculated and used to adjust for this random error, hence obtaining an improved estimate of the relationship with health outcomes [11]. Finally, accuracy and bias of the lifestyle indicators could be assessed in order to recommend the best season to take a given measure [12].

The primary aim of this observational study was to examine between-season and within-week variation in physical activity, sedentary behaviour, CRF and sleep duration in a large group of 8–11 year-old Danish school children. Additionally, we report the level of these lifestyle indicators, identify correlates of the lifestyle indicators and investigate the connection between them.

## Methods

### Study population

The initial sample comprised 834 of the 1021 invited 3rd and 4th grade students (8–11 years-old) from the nine representative Danish municipal schools enrolled in the OPUS (Optimal well-being, development and health for Danish children through a healthy New Nordic Diet) school meal study running from August 2011 to June 2012. The two primary outcomes of the OPUS project were to investigate concentration performance and metabolic health of a new Nordic diet served in school [13]. It is a cluster-randomised cross-over study with a number of measurements performed at baseline, at month 3 by the end of the first dietary period, and at month 6 by the end of the second dietary period. The nine schools were included from late August to November 2011. The three measurements separated by three months are used in the current paper, representing autumn (late August to November 2011), winter (November 2011 to February 2012) and spring (March to beginning of June 2012). The mean (mean of daily minimum and maximum) temperature [10.2 (7.2;13.0), 2.0 (−0.6;4.2) and 8.0 (4.5;11.7)°C], total rainfall (172, 208 and 112 mm) and sunshine (301, 229 and 575 hours) changed during autumn, winter and spring, respectively [14]. Furthermore, daylight increased by more than 10 hours from December to June. Children were excluded from the current analytical sample if they did not have valid measurements from all three seasons in either physical activity (≥10 hours of measured monitor wear time for at least 3 weekdays and 1 weekend day), CRF or sleep duration (at least 3 weekdays and 1 weekend day); hence, 730 children were included. The study was approved by The Committees on Biomedical Research Ethics for the Capital region of Denmark and written informed parental consent of both custody holders and child assent were obtained for all participants. The study was registered in the database http://www.clinicaltrials.gov (no. NCT01457794).

### Physical activity, sedentary time and sleep assessment

The children were asked to wear an ActiGraph™ accelerometer (GT3X + or GT3X) tightly at the right hip on an elastic belt for 7 consecutive days and 8 nights, and to remove it only during water activities (i.e. showering or swimming). At the end of the observation period, data were reintegrated to 60-second epochs and analysed using ActiLife6 (The ActiGraph 2012, ActiLife version 6).

Before analysis of physical activity and sedentary time we removed 1) data between midnight and 6 am as this was expected to be non-awake time, 2) periods of at least 15 minutes of consecutive zero counts using tri-axial vector magnitudes to remove non-wear time and non-awake time and 3) consecutive wear time periods of less than 60 minutes to remove non-awake time as sleep for most children is characterised by minor periods of movement that we did not want to include in our analysis of physical activity and sedentary time. Total physical activity (counts/min; cpm) was expressed as total vertical counts from wear-time, divided by measured wear-time. Time spent sedentarily was defined as all minutes showing 100 cpm or less, which is a widely used cut-off point [15]. Furthermore, moderate-to-vigorous physical activity (MVPA) was defined as ≥2296 cpm, which is a recently suggested pediatric cut-off point [15,16]. The number of children fulfilling the WHO recommendations of an average of ≥ 60 minutes MVPA/day [17] was also calculated. Weekly average of physical activity and sedentary time are calculated in the proportion 5 to 2 between weekdays (Monday-Friday) and weekend days (Saturday and Sunday).

The parents and children were instructed to keep logs for bedtimes (lights off and trying to sleep), and waking time (lights on) during the week in which the monitor was worn. To estimate accelerometer determined sleep duration, self-reported bedtimes and waking times were used as the possible window of sleep and accelerometer data within this window were scored in ActiLife6 using the algorithm by Sadeh et al. [18]. Self-reported sleep logs were missing for 4% (63 out of 1569) of the sleep measurements used in the current paper; in these cases, bedtimes and waking time were visually determined from the individual actograms. In a random subsample of 105 individuals we found the mean difference in sleep duration between these two approaches to be small (3.8 min, P < 0.001). The weekly average of sleep duration is calculated in the proportion 5 to 2 between weekdays (Sunday-Thursday) and weekend days (Friday and Saturday). In total, the accelerometer was worn for a median (interquartile range) duration of 21 (20–21) days and 23 (22–24) nights with at mean ± standard deviation (SD) wear-time of 900 ± 28 min/day between wake up time and bedtime.

### Cardio-respiratory fitness

Children ran as fast as possible between two parallel lines separated by 20 m for 15 sec following 15 sec of rest. This procedure was followed for 10 min and the total distance travelled was used to estimate the maximal oxygen uptake (VO_2max_) using an age-specific equation [19]: VO_2max_(ml/kg/min) = 18.17 + (0.03301 × distance)–(5.53 × gender) (boy = 0; girl = 1). Validity and reliability of this Andersen test were r = 0.64 and r = 0.84, respectively [19]. The test was performed at the schools by the physical education teachers and was supervised by scientific staff during the first of the three tests.

### Anthropometric measurements

Barefooted and wearing light clothes, the children were weighed to the nearest 0.1 kg (Tanita BWB-800S, Tokyo, Japan) and their heights were measured three times to the nearest 0.1 cm (average used) (CMS Weighing Equipment LTD, London, UK). BMI Z-score was calculated based on the WHO Growth Reference from 2007 [20] and the prevalence of underweight, normal weight, overweight and obese children was calculated based on age- and gender-specific cut-offs defined to pass through body mass index at 18.5, 25 and 30 kg/m^2^ at age 18 years [21,22].

### Questionnaire data

A baseline questionnaire ascertained age, gender, grade, highest education of household (divided into four groups according to years of education: ≤10 years, 11–12 years, 13–16 years, ≥17 years), number of parents born in Denmark (a proxy for ethnicity), and screen time. Screen time was computed based on the parent-reported time spent watching television, playing non-active video games or using the computer for leisure on weekdays and weekend days. Playing Nintendo Wii or similar active video game devices was not included in screen time. The weekly average of screen time was calculated in the proportion 5 to 2 between weekdays and weekend days. The number of children fulfilling the recommendations of less than two hours of screen time/day [23] was also calculated. Pubertal status was reported by parents and children in collaboration on the basis of breast development among girls and pubic hair among boys [24].

### Statistical analysis

Descriptive characteristics of the study sample are presented as mean ± SD or as proportions (%) and gender differences tested using a two-sample t-test or Pearson’s chi-squared test. A two-way ANOVA was carried out to investigate the effect of the diet intervention and season on all variables of interest. As no effect of the diet intervention was found in any of the variables (P > 0.33) the control- and intervention groups from the original study were combined for this study to maximize power. A one-way ANOVA with Bonferroni adjustments was carried out to investigate the effect of season, and ICC were calculated. To address the issue of accuracy, the mean absolute percentage error (MAPE) and bias were calculated for all lifestyle indicators between each season and average of the three measurements obtained during the different seasons as presented elsewhere [12]. To investigate the potential difference between weekdays and weekend days and the weekend day/weekday-ratio between seasons a paired t-test and Pearson’s chi-squared test was used. Non-normally distributed data were presented as median (interquartile range) and square-root-transformed before testing. The overall effect between variables of interest and season or weekdays/weekend days were not affected by gender (except sleep duration between weekdays and weekend days) and data for boys and girls were therefore combined. Univariate regression analysis was used to identify correlates of MVPA, sedentary time, CRF and sleep duration. Data are presented as mean and 95% confidence interval (CI). Linearity between residuals and the dependent variables in the model were visually checked along with normal distribution of the residuals and homogeneity of variance of the residuals. Partial correlation coefficients (adjusting for age, grade, gender and pubertal status) were performed between several lifestyle indicators. The level of significance was set at P < 0.05 and analyses were done using STATA/IC 11.2 (Houston, USA).

## Results

Descriptive characteristics of the participants are presented in Table 1. Overall, boys were slightly older than girls (P = 0.001) and more girls than boys had entered puberty (P < 0.001). A total of 13.3% of the children were classified as overweight or obese. As shown in Table 2, the children had 5% more sedentary time, 23% less time in MVPA and 2% longer sleep duration during winter compared to spring and CRF was 4% higher during spring compared to autumn (P < 0.001). Except from sedentary time, bias and MAPE were largest during spring. No differences were found in the variables measured at the first occasion with the inclusion criteria of having all three measurements (P > 0.21), except for sleep duration, which was 8 (95%CI 4–12) min less for the 247 children who were only measured at the first visit (P < 0.001).

**Table 1:** Descriptive characteristics of the study population by gender

**Variable**	**Boys (n = 376)**	**Girls (n = 354)**	**All (n = 730)**
Age (years)	10.0 ± 0.6	9.9 ± 0.6 *	10.0 ± 0.6
3rd grade/4th grade	49% / 51%	49% / 51%	49% / 51%
Pubertal status (% 1/2/≥3)	76.5 / 19.4 / 4.2	54.5 / 36.2 / 9.3 **	65.7 / 27.6 / 6.7
Parents born in Denmark (% 0 / 1 / 2)	11.2 / 12.5 / 76.3	10.5 / 11.9 / 77.7	10.8 / 12.2 / 77.0
Highest education of household (%)^1^	4.5 / 31.7 / 40.5 / 23.2	5.9 / 30.9 / 40.5 / 22.7	5.2 / 31.3 / 40.5 / 22.9
Weight (kg)	35.5 ± 7.2	34.5 ± 6.7	35.0 ± 7.0
Height (cm)	142.6 ± 7.1	142.1 ± 7.0	142.4 ± 7.1
Body mass index Z-score^2^	0.23 ± 1.12	0.04 ± 1.03 *	0.13 ± 1.08
Weight status (% uw/nw/ow/ob)^3^	8.3/77.8/11.5/2.4	12.3/75.1/11.4/1.1	10.2/76.5/11.5/1.8

Data are presented as mean ± standard deviation or proportions (%) and gender differences were tested using a two-sample t-test or Pearson’s chi-squared test. *, P < 0.05; **, P < 0.001.

^1^Highest education of household: ≤10 years / 11–12 years / 13–16 years / ≥17 years.

^2^Based on World Health Organization Growth Reference data from 2007 [20].

^3^Based on age- and gender-specific cut-offs defined to pass through body mass index at 18.5, 25 and 30 kg/m^2^ at age 18 years [21,22]; uw/nw/ow/ob, underweight/normal weight/overweight/obese.

**Table 2:** Physical activity, sedentary behaviour, sleep duration and cardio-respiratory fitness according to season of measurement in 8–11 year old Danish children

**Variables**	**n**	**Autumn**	**Winter**	**Spring**	**ICC**	**Bias (%)**	**MAPE (%)**
Sedentary time (min/d)	635	473 ± 63^a^	486 ± 59^b^	465 ± 66^a^ **	0.60	−0.4/2.5/-2.1	5.4/5.5/5.8
Screen-time (min/d)^1^	704	146 (102;193)					
Screen-time (≤2 h/d)^1,2^	704	34%					
Total PA (cpm)	635	490 ± 135^a^	447 ± 120^b^	557 ± 175^c^ **	0.47	−1.2/-9.9/11.1	11.6^a^/13.1^b^/15.1^c^ **
MVPA (min/d)	635	49 ± 23^a^	44 ± 22^b^	57 ± 27^c^ **	0.64	−2.0/-11.1/13.1	17.9^a^/19.4^ab^/21.1^b^ *
MVPA (≥1 h/d) (%)^3^	635	28%	22%	39% **			
CRF (ml/kg/min)^4^	492	47.5 ± 5.1^a^	48.3 ± 5.4^ab^	49.2 ± 5.8^c^ **	0.74	−1.6/-0.1/1.7	3.7^a^/3.2^b^/3.9^a^ *
Sleep duration (min/night)	523	556 ± 27^a^	553 ± 28^a^	541 ± 30^b^ **	0.50	1.2/0.5/-1.6	2.3^a^/2.2^a^/2.7^b^ **

*Abbreviations:ICC* Intraclass correlation coefficient, *MAPE* Mean absolute percentage error, *PA* Physical activity, *MVPA* Moderate-to-vigorous physical activity, *CRF* Cardio-respiratory fitness.

Data are presented as mean ± standard deviation or median (interquartile range) tested using one-way ANOVA with post-hoc Bonferroni test (non-normal distributed data was transformed before analysis) or proportions (%) tested using Pearson’s chi-squared test.

*, P < 0.05; **, P < 0.001. Different superscripts in a row (a,b,c) indicate significant differences (P <0.05).

^1^Only measured at baseline.

^2^Screen time recommendations [23].

^3^Physical activity recommendations [17].

^4^Distance run in the CRF-test at autumn, winter and spring were 973, 996 and 1023 meters, respectively.

As shown in Table 3 sedentary time and screen-time were higher and total physical activity, MVPA and sleep duration (only boys) were lower during weekends at all measurement occasions (P ≤ 0.01). The weekend day/weekday-ratio of total physical activity and MVPA was lower during winter compared to spring (P < 0.001).

**Table 3:** Physical activity, sedentary behaviour, sleep duration and cardio-respiratory fitness at weekdays and weekend days at three seasons in 8–11 year old Danish children

**Variables**	**n**		**Autumn**			** Winter**			** Spring**	
		**Weekday**	**Weekend**	**Ratio**^**5**^	**Weekday**	**Weekend**	**Ratio**^**5**^	**Weekday**	**Weekend**	**Ratio**^**5**^
Sedentary time (min/d)	635	467 ± 66	488 ± 88**	1.05	477 ± 61	509 ± 82**	1.07	459 ± 67	481 ± 94**	1.06
Screen-time (min/d)^1^	704	120 (80;165)	210 (135;300)**							
Screen-time (≤2 h/d)^1,2^	704	46%	17%**							
Total PA (cpm)	635	507 (417;614)	400 (295;514)**	0.84^a^	464 (388;566)	347 (259;441)**	0.78^b^	555 (450;671)	460 (331;635)**	0.92^c^ **
MVPA (min/d)	635	50 (34;71)	28 (16;47)**	0.68^a^	46 (32;66)	24 (12;39)**	0.63^a^	56 (38;78)	40 (21;62)**	0.83^b^ **
MVPA (≥1 h/d)^3^	635	37%	15%**	0.41	33%	9%**	0.28	45%	27%**	0.60 *
Sleep duration (min/night)^4^	523	559 ± 28	549 ± 48**	0.99	554 ± 31	549 ± 48*	1.00	543 ± 32	536 ± 55*	0.99

*Abbreviations:PA* Physical activity, *MVPA* Moderate-to-vigorous physical activity.

Data are presented as mean ± standard deviation or median (interquartile range) tested by student t-test or one-way ANOVA with post-hoc Bonferroni test (non-normal distributed data were transformed before analysis) or proportions (%) tested by Chi-square test. *, P < 0.05; **, P < 0.001. Different superscripts in a row (a,b,c) indicate significant difference (P ≤0.05).

^1^Only measured at baseline.

^2^Screen time recommendations [23].

^3^Physical activity recommendations [17].

^4^Boys were sleeping 12, 11 and 13 min/night less during weekend days compared to weekdays in autumn, winter and spring (P < 0.001). Girls were not sleeping less during weekend days compared to weekdays (3, -4 and 0 min/night in autumn, winter and spring (P ≥ 0.22).

^5^Weekend day/weekday-ratio within season.

As shown in Table 4, an increase in age of 1 year was associated with a decrease in MVPA (2.7 min/day) and sleep duration (10.2 min/night) as well as an increase in sedentary time (27.8 min/day) and CRF (0.8 ml/kg/min) (P < 0.05). The same pattern was seen for the difference between 3rd and 4th graders, except for CRF, with 4th graders accumulating 5.4 min/day less MVPA, being 32.4 min/day more sedentary and sleeping for 13.6 min less (P < 0.001). Children who had entered puberty (Tanner stage ≥2) had 7.5 min less MVPA, 12.0 min/day more sedentary time, 7.2 min/night less sleep and a 2.5 ml/kg/min lower CRF compared to those who had not (Tanner stage =1) (P < 0.05), while boys had 19.9 min/day more MVPA, 5.1 min/night less sleep and an 8.0 ml/kg/min higher CRF compared to girls (P < 0.05). Children with two parents born outside Denmark had 19.2 min/night less sleep compared to children having both parents born in Denmark (P < 0.05). Children whose parents had the lowest education (≤10 years) were sleeping for approximately 24 min less compared to all other educational groups, and were roughly 24 min less sedentary than children whose parents had an education of ≥13 years (P < 0.05). Adjusting wear time for sedentary time, total physical activity and MVPA or expressing sedentary time and MVPA as percentage of wear time did not change the results from the regression analysis (except for sedentary time that was 16.6 min/day lower in children having two parents born outside Denmark compared to no parents born outside Denmark).

**Table 4:** Associations between average of the three seasons of moderate-to-vigorous physical activity, sedentary time, sleep duration and cardio-respiratory fitness with potential explaining variables in 8–11 year old Danish children

**Variable**^**1**^	**n**	**Moderate-to-vigorous physical activity (min/day) (n = 635)**		**Sedentary time (min/day) (n = 635)**		**Sleep duration (min/night) (n = 523)**		**Cardio-respiratory fitness (ml/kg/min) (n = 492)**	
		**B (95%CI)**	***P***	**B (95%CI)**	***P***	**B (95%CI)**	***P***	**B (95%CI)**	***p***
Age (years)	730	−2.7 (−4.5;-0.8)	0.046^3^	27.8 (21.5;34.1)	<0.001	−10.2 (−13.4;-7.1)	<0.001	0.8 (0.1;1.5)	0.03
Grade	730								
3rd graders	359	Reference		Reference		Reference		Reference	
4th graders	371	−5.4 (−8.7;-2.1)	<0.001	32.4 (24.4;40.4)	<0.001	−13.6 (−17.5;-9.6)	<0.001	−0.1 (−1.0;0.8)	0.87
Pubertal status	706								
1	464	Reference		Reference		Reference		Reference	
≥2	242	−7.5 (−11.0;-3.9)	<0.001	12.0 (3.0;21.0)	0.01	−7.2 (−11.6;-2.9)	0.001	−2.5 (−3.4;-1.6)	<0.001
Gender	730								
Girls	354	Reference		Reference		Reference		Reference	
Boys	376	19.9 (16.9;22.9)	<0.001	−0.3 (−8.7;8.1)	0.95	−5.1 (−9.2;-1.0)	0.01	8.0 (7.5;8.5)	<0.001
Parents born in Denmark^2^	730								
0	79	Reference		Reference		Reference		Reference	
1	89	2.3 (−4.8;9.4)	0.52	12.6 (−5.2;30.4)	0.16	20.8 (11.2;30.5)	<0.001	1.3 (−0.7;3.2)	0.20
2	562	−2.4 (−8.0;3.2)	0.40	13.5 (−0.4;27.4)	0.06^4^	19.2 (11.1;30.5)	<0.001	0.1 (−1.5;1.7)	0.89
Highest education of parents^2^	728								
≤10 years	38	Reference		Reference		Reference		Reference	
11 - 12 years	228	−3.1 (−11.6;5.3)	0.46	13.0 (−7.9;34.0)	0.22	24.4 (12.9;35.9)	<0.001	0.8 (−1.3;3.0)	0.45
13 - 16 years	295	−4.0 (−12.2;4.3)	0.35	22.5 (1.9;43.1)	0.03	26.0 (14.7;37.4)	<0.001	1.1 (−1.1;3.3)	0.31
≥17 years	167	−1.3 (−9.9;7.2)	0.76	24.0 (2.7;45.3)	0.03	23.0 (11.4;34.6)	<0.001	1.5 (−0.7;3.7)	0.20

^1^All dependent variables are from the baseline assessments.

^2^P > 0.05 between non-reference groups.

^3^B = −2.4 (95%CI = −5.8;1.0, P = 0.17) among children with a valid cardio-respiratory fitness test (n = 413).

^4^B = 16.6 (95%CI = 3.4;29.9, P = 0.01) if wear-time was included as covariate.

Partial correlations were carried out between physical activity, sedentary time, screen time, sleep duration and CRF, with adjustment for age, grade, gender and pubertal status, from baseline and from an average of the three seasons (Table 5). Partial correlation coefficients numerically increased between CRF and each of sedentary time, total physical activity and MVPA when using the average of the three seasons compared to baseline only. Moderate to strong correlations (0.49-0.91) were seen between sedentary time, total physical activity and MVPA, with MVPA explaining 82.8% of the variance in total physical activity. Sleep was negatively correlated with screen time (r = −0.15, P = 0.001), total physical activity (r = −0.15, P = 0.001) and MVPA (r = −0.18, P < 0.001), but not with sedentary time. Screen time was also negatively correlated with total physical activity (r = −0.11, P = 0.01) and CRF (r = −0.16, P < 0.001), but positively correlated with sedentary time (r = 0.17, P < 0.001).

**Table 5:** Partial correlation coefficients (r) between sedentary time, screen time, physical activity, cardio-respiratory fitness and sleep duration from baseline and average of the three seasons

	**Sedentary time (min/day)**	**Screen time (min/day)**	**Total PA (cpm)**	**MVPA (min/day)**	**CRF (ml/kg/day)**
Screen time (min/day)^1^	0.18 / 0.17				
Total PA (cpm)	−0.70 / -0.69	−0.13 / -0.11			
MVPA (min/day)	−0.49 / -0.49	−0.08 / -0.06	0.89 / 0.91		
CRF (ml/kg/day)	−0.05 / -0.17^2^	−0.14 / -0.16	0.22 / 0.45	0.29 / 0.50	
Sleep duration (min/night)	0.06 / 0.05	−0.14 / -0.15	−0.13 / -0.15	−0.15 / -0.18	0.02 / -0.05

*Abbreviations:PA* Physical activity, *MVPA* Moderate-to-vigorous physical activity, *CRF* Cardio-respiratory fitness.

First number is partial correlation coefficients (r) from baseline measurements only. Second number is r from the average of all three seasons. Both are controlled for age, grade, gender and pubertal status.

r ≥ |0.09|: P < 0.05.

r ≥ |0.16|: P < 0.001.

^1^Screen time is only measured at baseline.

^2^When adjusted for MVPA: r = 0.08; p = 0.09.

## Discussion

Overall, sedentary time was higher and physical activity, including the number of children fulfilling the physical activity recommendations, was lower during winter and during weekends. Sleep duration was shorter during spring and among boys also during weekends. Younger boys who had not entered puberty were more physically active, less sedentary and had higher CRF, whereas having two parents born outside Denmark or with a low education were associated with shorter sleep but also with less time spent on sedentary activities.

### Level of physical activity, sedentary behaviour, sleep and fitness

The higher CRF among boys compared to girls was more pronounced than previously reported from a comparable Danish population using the same fitness test protocol [25], but similar to children at the same age from a large European study using a cycle test [1]. For most individuals (ranging from 61 to 78% in the different seasons) MVPA was lower than recommended, which is comparable to other recent studies using 60 seconds epochs despite small methodological differences (e.g. cut-off and wear time criteria) [26,27]. Boys had a 50% higher MVPA compared to girls, which could have contributed to the large gender difference in CRF. Sedentary time was two hours longer than recently reported in a pooled analysis of more than 20,000 4–18 year old children [3]. The fact that we used a 24-hour accelerometer protocol is likely to have contributed to the relatively high amount of time spent in a sedentary state and relatively low amount of physical activity compared to other studies. A recent large European study, however, found an even higher amount of time spent sedentary and lower physical activity in an adolescent population without using a 24-hour accelerometer protocol [27]. Screen time use was high in the present study, with the majority exceeding the recommendations (66%). Screen time was less than previously reported in a study from Canada [28], but comparable to earlier studies from Denmark [29]. According to the National Sleep Foundation, school-aged children between 5 and 12 years need a minimum of 10 hours of sleep per night [30]. The reported “time in bed” in the present study was 10 hours (data not shown), and other studies using parent or self-reported sleep duration measures have similar findings [31,32]. Using the objective measurement of sleep, however, sleep duration was lower (9 hours and 10 min), indicating an insufficient amount of sleep. This suggests that objective measures of lifestyle indicators are needed if we really want to have an accurate picture.

### Between-season and within-week variation in physical activity, sedentary behaviour, sleep and fitness

Cardio-respiratory fitness fluctuated less within individuals during a school year (highest ICC) compared to sedentary time, physical activity and sleep duration, as might be expected. Using accelerometers most studies find children of approximately the same age as the present study to be most physically active during the summer [7,8,33-37] or (if summer is not measured) spring [38-41]; however, a couple of studies found no seasonal variation [9,42], and a decrease in outdoor activity has even been observed during summer in a hot and humid area [43]. In the current study, ICC from total physical activity was lower (0.47 vs. 0.54 and 0.63 in boys and 0.86 in girls) and MVPA intermediate (0.64 vs. 0.51 and 0.68 in boys and 0.72 in girls) compared to the two other studies that have examined seasonal change in physical activity of children using a repeated-measure design and reported ICC [7,8]. Studies looking at seasonal changes in sedentary time using accelerometers are sparse, inconsistent, and cross-sectional of nature [34,35,42]. Two studies from the UK in 5 and 7 year old children, respectively, found less sedentary time during summer compared to the rest of the year or more sedentary time during spring compared to summer and autumn [34,35]. Finally, sedentary time has been found not to be influenced by season of measurement [42]. In our study, it seems that sedentary time is inversely related to MVPA. The same reliability (ICC) has been reported previously, however, without reporting the actual level of sedentary time [7]. Seasonal variations in CRF vary widely from one study to another [44], and could, in part, depend on the method used and the timing of assessment. We observed a higher CRF during spring compared to autumn, which is in good agreement with the higher total physical activity and MVPA seen during spring. However, as there was a tendency for children to run longer in a second CRF test in the validation paper (+15 m ≈ 0.5 ml/kg/min, P = 0.10; n = 47) [19], a learning effect could partly explain the higher CRF during spring in the present study, as it was the last test that was performed. Furthermore, as children become 6 month older from autumn to spring, the higher CRF during spring may also partly be the result of increasing age rather than seasonal variation. We found sleep duration to be shorter during spring compared to autumn and winter. The same was found in a cross-sectional study of 7 year-old children using accelerometers; however that study indicated that sleep duration was even lower during summer [45]. This shorter sleep duration during spring has also been reported in university students [46] and under conditions of temporal isolation, where subjects can choose their sleep duration [47].

Given that hours of daylight and sunshine were lowest and rainfall highest during the winter, followed by autumn and spring, there is good reason to believe that the differences found in the present study could be caused by the changing weather conditions of a northern European country. As children were more physically active during spring compared to autumn, our data suggest that rainfall and hours of daylight and sunshine have a larger impact on physical activity level compared to a modest change in temperature. This is supported by a study showing that regardless of season Canadian adolescents were found to have 1-2% higher physical activity for every 10°C increase in temperature, but 2-4% lower physical activity for every 10 mm of rainfall [48]. Furthermore, the most likely explanation of the decreased sleep duration during spring compared to autumn and winter is the longer photoperiod at high northern or southern latitudes. Collectively, in the present study, differences in lifestyle indicators between seasons were found. Given that the weather conditions change within seasons (although to a minor extent), variation in lifestyle indicators within each season could be anticipated as previously reported in Canadian adolescents using physical activity recalls [48]. All lifestyle indicators differed between weekdays and weekend days at all three seasons (except sleep duration among girls). The lower physical activity during weekends was especially evident during winter. As winter in Denmark is characterised by being cold, rainy and dark one might propose that physical activity level of the children could benefit from providing more indoor alternatives to the MVPA accumulated outdoor during the rest of the year.

The relatively high intra-individual variation could lead to random error when a single measure (e.g. physical activity) from a random time during the year is collected to quantify a relationship with obesity or cardio-metabolic risk. However, when repeated measurements of the exposure variable (e.g. physical activity) are collected, the ICC which is a measure of reliability can be calculated and used to adjust for this random error, hence obtaining an improved estimate of the relationship with health outcomes. Based on our calculated bias and MAPE, estimates from spring were the most inaccurate and biased estimates of habitual lifestyle whereas autumn were the most accurate and unbiased estimates of habitual physical activity.

### Correlates of physical of activity, sedentary behaviour, sleep and fitness

Collectively, MVPA and sleep duration were lower and sedentary time higher for older individuals. Given that the need for sleep decreases from childhood to adulthood, this observation seems reasonable; however, as time spent in a sedentary state was markedly higher in older individuals, it is unclear whether this shorter sleep duration is due to a decreased need or to changed behaviour of the older children. Cardio-respiratory fitness was positively associated with age, but negatively associated with pubertal stage. As CRF is expressed per kg bodyweight and puberty is a time of rapid weight gain, this negative association seems reasonable. Finally, we speculated that the lifestyle indicators could be different among children with different ethnicity and education and found that, having two parents born outside Denmark or with low education levels was associated with shorter sleep duration and less time spent in a sedentary state. As these children did not accumulate more MVPA, data suggest that they accumulated more light physical activity as they did not have less wear-time (data not shown).

### Connections between physical activity, sedentary behaviour, sleep and fitness

Most of the correlations between the above variables were rather weak (r < 0.20); however, total physical activity, MVPA and sedentary time had moderate to high correlations (r between 0.49-0.91). The relatively strong correlations between these variables may partly be considered a methodological matter, as they are a part of the same 24 hours, and partly a true observation. These variables are difficult to separate from each other; however, our negative correlations fit well with existing literature in children [3]. Our negative correlation observed between sleep duration and total physical activity (and MVPA) is supported by a recent study that proposed the inherent activity level to manifest itself in high activity level both during the day and the night [49], which by definition results in shorter accelerometer determined sleep duration. Furthermore, the negative correlation between sleep duration and screen time, but not between sleep duration and sedentary time, could indicate that delayed sleep onset was caused by extensive screen time in the later part of the evening among certain individuals [50]. Finally, the correlations became stronger between CRF and each of sedentary time, total physical activity and MVPA when including an average of the three seasons. Given that total physical activity and in particular MVPA are expected to correlate relatively closely with CRF, the increase from 8 to 25% of the explained variation between MVPA and CRF indicate the strength of having multiple measures during various seasons and including both weekdays and weekend days.

### Strength and limitations

In a school-based study it is often difficult to differentiate the effect of school or school neighbourhood on physical activity from the effect of season because different children and schools are included at different points in time. As children from all schools were measured during all three seasons our observed effect of season was independent of school and school neighbourhood. A further strength of the study is the assessment of repeated measures across different seasons, which makes it possible to determine ICC, bias, MAPE and minimise random error when used as potential explanatory variables. Finally, this paper introduce new standards by reporting the amount of time spent being sedentary, active and sleeping in a large cohort of children studied in their natural environment with the use of objective measurements.

Limitations of the measure of physical activity used are the inability to capture cycling and load-bearing activity as well as swimming and other activities where the monitor was removed. Bearing this in mind, and the fact that the PA recommendations are based on studies using self-reported data which tend to overestimate activity, the number of children fulfilling the physical activity recommendations might be underestimated in this and other studies that use accelerometers. As self-reported time spent cycling during weekdays was lower during winter (10 min/day) compared to autumn and spring (14 min/day; P < 0.001) (data not shown) our observed differences in physical activity between seasons are expected to be even larger than reported. Time spent ≥1520 cpm have been shown not to be influenced by epoch length in adolescents and time spent ≥3450 to be underestimated in children using 60 sec epochs compared to 5, 15 and 30 sec [51]. It is, therefore, possible that MVPA (time spent ≥2296 cpm) in our study is underestimated; however to compare with previously conducted studies and to use validated cut-off points, an epoch length of 60 sec was used from the vertical axis only. We did not separately examine moderate PA and vigorous PA as accelerometers have a limited capacity to correctly classify these when using an epoch length of 60 sec [51]. No overall differences were observed if total physical activity was expressed as 3-axis vector magnitude and the seasonal and weekly variation in lifestyle indicators were not affected by wear-time. In a recent pilot-study using a similar population we tested the accordance between waist- and wrist-worn devices and concluded that waist-worn devices can provide a proxy measure of sleep duration in epidemiologic studies [52]. The correlations and differences between seasons could, therefore, be trusted; however, given that waist-worn devices tend to overestimate sleep compared to wrist-worn devices [52], the actual sleep duration in the present study might be shorter than reported. Other things to bear in mind is that the educational level of the parents was somewhat higher than the average Danish population, and the fact that no measurements were taken during the summer, since this was a school based-study collecting data during normal school weeks and not during vacations.

## Conclusions

Approximately two thirds of the sample exceeded the screen time recommendation of <2 h per day and two thirds did not fulfil the physical activity recommendation of 60 min MVPA per day. Given that sedentary time was higher and physical activity (including number of children fulfilling physical activity recommendations) was lower during winter and during weekends, these time-periods might serve as key intervention periods in order to promote a healthy lifestyle in countries at high northern latitudes. The most accurate and unbiased estimates of physical activity came from autumn; however children’s sedentary time, physical activity, sleep duration and CRF showed considerable intra-individual variation when measured across three different seasons, suggesting that a single measurement taken at one point in time may not adequately provide an accurate assessment of these lifestyle indicators in children.

## Abbreviations

CI: Confidence interval; CPM: Counts/min; CRF: Cardio-respiratory fitness; ICC: Intra-class correlation coefficient; MVPA: Moderate-to-vigorous physical activity.

## Competing interests

The authors declare that they have no competing interests.

## Authors’ contributions

MH coordinated the data collection, analysed and interpreted the data and drafted the manuscript. AA, IT and KM were active in the planning of the study and reviewed the article critically. AS were active in the planning of the study, discussed the analysis and interpretation of the data and reviewed the manuscript critically. JP discussed the analysis and interpretation of the data and reviewed the manuscript critically. All authors read and approved the final manuscript.

## Pre-publication history

The pre-publication history for this paper can be accessed here:

http://www.biomedcentral.com/1471-2458/13/808/prepub
